# Puumala Virus in Bank Voles, Lithuania

**DOI:** 10.3201/eid2301.161400

**Published:** 2017-01

**Authors:** Petra Straková, Sandra Jagdmann, Linas Balčiauskas, Laima Balčiauskienė, Stephan Drewes, Rainer G. Ulrich

**Affiliations:** Academy of Sciences, Brno, Czech Republic (P. Straková);; Masaryk University, Brno (P. Straková);; Friedrich-Loeffler-Institut, Greifswald-Insel Riems, Germany (P. Straková, S. Jagdmann, S. Drewes, R.G. Ulrich);; Nature Research Centre, Vilnius, Lithuania (L. Balčiauskas, L. Balčiauskienė)

**Keywords:** Puumala virus, viruses, bank voles, Myodes glareolus, zoonoses, Lithuania

## Abstract

Little is known about the presence of human pathogenic Puumala virus (PUUV) in Lithuania. We detected this virus in bank voles (*Myodes glareolus*) in a region of this country in which previously PUUV-seropositive humans were identified. Our results are consistent with heterogeneous distributions of PUUV in other countries in Europe.

Puumala virus (PUUV) (family *Bunyaviridae*) is an enveloped hantavirus that contains a single-stranded trisegmented RNA genome of negative polarity (*1*). PUUV harbored by the bank vole (*Myodes glareolus*) is the most prevalent human pathogenic hantavirus in Europe (*2*). A high population density of bank voles can lead to disease clusters and possible outbreaks of nephropathia epidemica, a mild-to-moderate form of hantavirus disease (*3*).

In contrast to the Fennoscandian Peninsula and parts of central Europe (*4**,**5*), little is known about the epidemiology of PUUV in Poland and the Baltic States. Recent investigations confirmed the presence of PUUV in certain parts of Poland (*5**,**6*). A molecular study of bank voles in Latvia identified 2 PUUV lineages (Russian and Latvian) (*7*). In Estonia, serologic and molecular screening provided evidence of the Russian PUUV lineage (*8*). For Lithuania, a previous serosurvey indicated the presence of PUUV-specific antibodies in humans from 3 counties (Technical Appendix Figure 1). However, molecular evidence of PUUV in humans or in voles is lacking (*9*).

We report a molecular survey of rodent populations in Lithuania at 5 trapping sites, including 2 sites in counties where PUUV-specific antibodies were previously detected in humans (Technical Appendix Figure 1). A total of 134 bank voles, 72 striped field mice (*Apodemus agrarius*), and 59 yellow-necked field mice (*A. flavicollis*) were captured during 2015. Three trapping sites (Juodkrantė, Elektrėnai, and Lukštas) were located in forests at or near a cormorant colony, and 2 trapping sites (Žalgiriai and Rusnė) were located in a wet forest and flooded meadows. All applicable institutional and national guidelines for the care and use of animals were followed.

For PUUV detection, we extracted RNA from bank vole lung tissue samples by using the Qiazol Protocol (QIAGEN, Hilden, Germany) and conducting screening by using a small segment RNA–specific reverse transcription PCR (RT-PCR) and primers Pu342F and Pu1102R (*6*). We detected PCR products for 5 (LT15/164, LT15/165, LT15/166, LT15/174, and LT15/201) of 45 bank voles from the Lukštas trapping site. All 9 striped field mice and 2 yellow-necked field mice from Lukštas showed negative results for the PUUV RT-PCR.

We amplified the complete nucleocapsid protein–encoding region for 3 of the 5 samples positive by RT-PCR with 3 primer pairs: PuNCRS (5′-TAGTAGTAGACTCCTTGAA-3′)/Pu255R (5′-TGGACACAGCATCTGCCA-3′), Pu40F (5′-CTGGAATGAGTGACTTAAC-3′)/Pu393R (5′-TATGGTAATGTCCTTGATGT-3′), and Pu1027F (5′-ATGGCAGAGTTAGGTGCA-3′)/Pu1779R (5′-TCAGCATGTTGAGGTAGT-3′). RT-PCR products were directly sequenced by using the BigDye Terminator Version 1.1 Cycle Sequencing Kit (Applied Biosystems, Darmstadt, Germany). We deposited the sequences of the 5 samples in GenBank under accession nos. KX757839, KY757840, KX 757841, KX751706, and KX751707 (Figure; Technical Appendix Figure 2).

**Figure:**
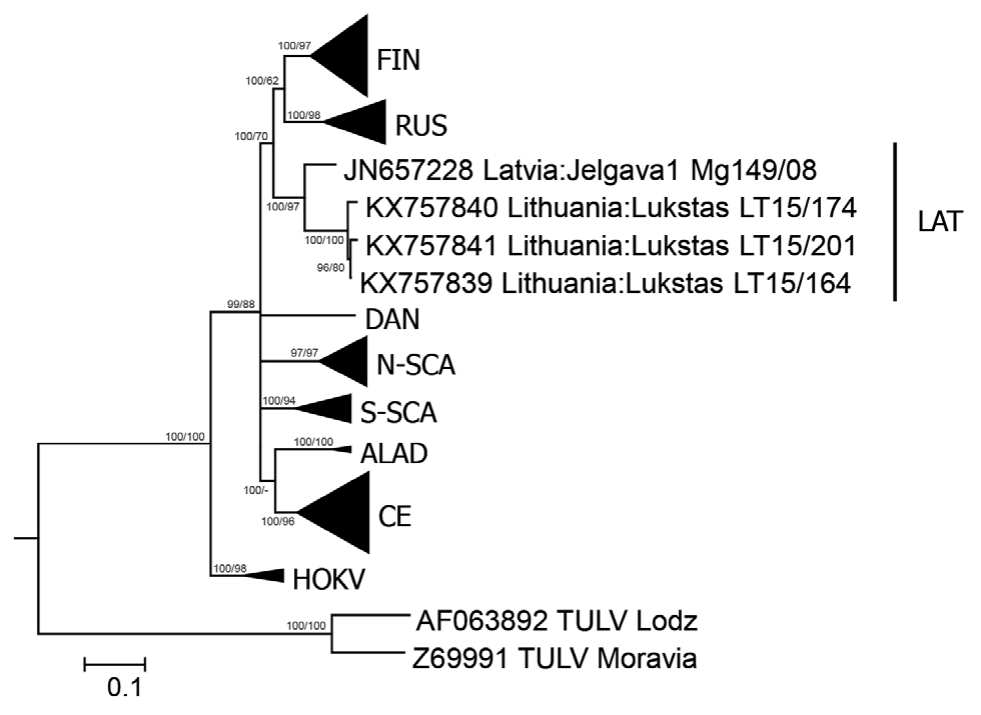
Phylogenetic tree based on complete nucleocapsid gene sequences of Puumala virus (PUUV) strains from Lithuania (LT), Latvia (Jelgava1), and other PUUV clades. Tula virus (TULV) was used as the outgroup. The tree was generated by Bayesian and maximum-likelihood analysis using MrBayes 3.2.6 (http://mrbayes.sourceforge.net/download.php) and MEGA6 software (http://www.megasoftware.net/). The optimal substitution model was calculated by using jModelTest 2.1.4 (https://code.google.com/p/jmodeltest2). The Bayesian tree was based on transition model 2 with invariant sites and gamma distribution and 4 million generations. For maximum-likelihood analysis, the Kimura 2-parameter model and 1,000 bootstrap replicates were used. Posterior probabilities are indicated before slashes, and bootstrap values are indicated after slashes. Scale bar indicates nucleotide substitutions per site. ALAD, Alpe-Adrian lineage; CE, Central European lineage; DAN, Danish lineage; FIN, Finnish lineage; HOKV, Hokkaido virus; LAT, Latvian lineage; N-SCA, North-Scandinavian lineage; RUS, Russian linage; S-SCA, South-Scandinavian lineage.

The 3 nucleocapsid protein–encoding nucleotide sequences showed identities of 98.2%–99.8%, and the 3 deduced nucleocapsid protein amino acid sequences showed identities of 99.8%–100% (Technical Appendix Table). We found the highest similarity of the 3 nucleotide and corresponding amino acid sequences for the PUUV strain from Latvia (Jelgava1/Mg149/2008; JN657228): nucleotide sequence 89.8%–90.4% and amino acid sequence 99.8%–100% (Technical Appendix Table).

We generated phylogenetic trees by using MrBayes 3.2.6 software (http://mrbayes.sourceforge.net/download.php) and MEGA6 software (http://www.megasoftware.net/) for complete (1,302 nt; Figure) and partial (465 nt; Technical Appendix Figure 2) nucleocapsid protein–encoding sequences. Phylogenetic analysis confirmed results of pairwise nucleotide sequence divergence analysis, which indicated clustering of PUUV sequences from Lithuania with sequences from northern Poland (Technical Appendix Figure 2) and the Jelgava 1 strain from Latvia (Figure). These sequences of the Latvian clade are well separated from the Russian and all other European PUUV clades.

To evaluate a potential association of PUUV with evolutionary lineages of the bank vole, we determined vole cytochrome b gene sequences, deposited them in GenBank under accession nos. KX769843 (LT15/164), KX769844 (LT15/165), KX769845 (LT15/166), KX769846 (LT15/174), and KX769847 (LT15/201), and compared them with cytochrome b prototype sequences of evolutionary lineages. Consistent with results for northern Poland (*6*), we identified 2 bank vole lineages at Lukštas, and the PUUV sequences were detected in 4 bank voles of the Carpathian phylogroup and in 1 vole of the Eastern lineage.

In conclusion, we detected PUUV in bank voles at 1 site (Lukštas) in Lithuania (prevalence of 11.1%). This site is located in a region where PUUV-seropositive persons were identified (*9*) and near the border with Latvia (Technical Appendix Figure 1). The absence of PUUV in bank voles at 4 other sites might have been caused by the small number of voles tested. However, our results are consistent with heterogeneous distributions of PUUV in other countries (*10*).

Detection of this novel PUUV strain by using a specific RT-PCR confirms the reliability of this assay for molecular diagnostic and epidemiologic studies of this virus in Lithuania. Future large-scale monitoring studies are needed to evaluate the geographic distribution and temporal fluctuation of PUUV in bank vole populations in Lithuania.

Technical Appendix Additional information on analysis of Puumala virus in bank voles, Lithuania.

## References

[1] Plyusnin A, Beaty BJ, Elliot RM, Goldbach R, Kormelink R, Lundkvist A, et al. Family *Bunyaviridae*. In: King AM, Adams MJ, Carstens EB, Lefkowitz EJ, editors. Virus taxonomy: ninth report of the international committee on taxonomy of viruses. San Diego: Elsevier Academic Press; 2012. p. 725–41.

[2] Heyman P, Ceianu CS, Christova I, Tordo N, Beersma M, João Alves M, et al. A five-year perspective on the situation of haemorrhagic fever with renal syndrome and status of the hantavirus reservoirs in Europe, 2005-2010. Euro Surveill. 2011;16:19961. 21924118 10.2807/ese.16.36.19961-en

[3] Clement J, Maes P, van Ypersele de Strihou C, van der Groen G, Barrios JM, Verstraeten WW, et al. Beechnuts and outbreaks of nephropathia epidemica (NE): of mast, mice and men. Nephrol Dial Transplant. 2010;25:1740–6. 10.1093/ndt/gfq122 20237057

[4] Klempa B, Radosa L, Krüger DH. The broad spectrum of hantaviruses and their hosts in Central Europe. Acta Virol. 2013;57:130–7. 10.4149/av_2013_02_130 23600871

[5] Michalski A, Niemcewicz M, Bielawska-Drózd A, Nowakowska A, Gaweł J, Pitucha G, et al. Surveillance of hantaviruses in Poland: a study of animal reservoirs and human hantavirus disease in Subcarpathia. Vector Borne Zoonotic Dis. 2014;14:514–22. 10.1089/vbz.2013.1468 24902039 PMC4098853

[6] Ali HS, Drewes S, Sadowska ET, Mikowska M, Groschup MH, Heckel G, et al. First molecular evidence for Puumala hantavirus in Poland. Viruses. 2014;6:340–53. 10.3390/v6010340 24452006 PMC3917447

[7] Razzauti M, Plyusnina A, Niemimaa J, Henttonen H, Plyusnin A. Co-circulation of two Puumala hantavirus lineages in Latvia: a Russian lineage described previously and a novel Latvian lineage. J Med Virol. 2012;84:314–8. 10.1002/jmv.22263 22170553

[8] Golovljova I, Sjölander KB, Lindegren G, Vene S, Vasilenko V, Plyusnin A, et al. Hantaviruses in Estonia. J Med Virol. 2002;68:589–98. 10.1002/jmv.10231 12376968

[9] Sandmann S, Meisel H, Razanskiene A, Wolbert A, Pohl B, Krüger DH, et al. Detection of human hantavirus infections in Lithuania. Infection. 2005;33:66–72. 10.1007/s15010-005-4058-8 15827873

[10] Drewes S, Turni H, Rosenfeld UM, Obiegala A, Strakova P, Imholt C, et al. Reservoir-driven heterogeneous distribution of recorded human Puumala virus cases in South-West Germany. Zoonoses Public Health. 2016. In press. 10.1111/zph.12319 27918151

