# Correction: Vol. 23, No. 1

**DOI:** 10.3201/eid2304.C32304

**Published:** 2017-04

**Keywords:** errata, erratum, correction

The GenBank accession no. KX757840 was listed incorrectly in Puumala Virus in Bank Voles, Lithuania (P. Straková et al.). The article has been corrected online (https://wwwnc.cdc.gov/eid/article/23/1/16-1400_article).

