# Supplementary material for: Puumala Virus in Bank Voles, Lithuania
Source: Emerg Infect Dis. 2017 Jan;23(1):158–60. doi: 10.3201/eid2301.161400 (PMC5176220; doi:10.3201/eid2301.161400)
Supplement: Technical Appendix — Additional information on analysis of Puumala virus in bank voles, Lithuania. [file 16-1400-Techapp-s1.pdf]

Article DOI: http://dx.doi.org/10.3201/eid2301.161400

Puumala Virus in Bank Voles, Lithuania

Technical Appendix

Technical Appendix Table. Percent pairwise identity of small segment RNA nucleotide sequences (above the diagonal) and amino acid sequences of nucleocapsid protein (below the diagonal) for Puumala virus strains*

Strain	LT15/164	LT15/174	LT15/201	LAT	RUS1	RUS2	RUS3	FIN1	FIN2	N-SCA1	N-SCA2	S-SCA1	S-SCA2	CE1	CE2	DAN	ALAD
LT15/164		98.39	99.77	90.40	85.48	85.64	85.87	86.71	86.02	85.18	85.10	84.87	85.79	85.48	85.10	84.49	85.41
LT15/174	100.00		98.16	89.78	86.02	85.94	86.10	86.71	86.33	85.48	84.87	84.72	85.25	85.33	85.10	84.87	84.95
LT15/201	99.77	99.77		90.17	85.41	85.56	85.79	86.48	85.94	84.95	84.87	84.64	85.56	85.41	84.95	84.25	85.18
LAT	100.00	100.00	99.77		85.25	84.87	85.02	87.40	86.56	85.64	85.18	85.64	86.02	84.95	86.10	85.64	85.64
RUS1	96.77	96.77	96.77	96.77		95.47	86.48	84.49	85.10	84.72	83.33	84.25	84.72	82.03	83.72	82.64	83.72
RUS2	97.00	97.00	96.77	97.00	98.85		86.87	84.49	85.64	84.56	83.87	83.56	84.79	82.95	83.26	82.26	83.49
RUS3	96.77	96.77	96.54	96.77	96.54	97.23		85.64	84.95	83.87	84.56	83.18	84.49	83.64	83.56	83.72	84.18
FIN1	97.23	97.23	97.00	97.23	96.30	97.00	96.77		92.78	85.41	84.72	84.79	85.02	84.64	83.18	82.80	85.10
FIN2	97.00	97.00	96.77	97.00	96.07	96.54	96.77	98.61		84.10	83.79	84.79	84.87	84.41	83.26	82.95	85.18
N-SCA1	98.38	98.38	98.15	98.38	96.30	97.00	96.30	96.54	96.07		89.94	85.56	85.48	83.56	84.25	84.18	84.41
N-SCA2	98.15	98.15	97.92	98.15	95.61	95.84	95.15	95.84	95.61	98.38		84.79	83.87	83.87	84.18	84.49	83.72
S-SCA1	97.69	97.69	97.46	97.69	96.07	96.30	96.07	95.84	95.61	96.54	96.54		87.71	84.18	84.25	84.49	85.48
S-SCA2	98.61	98.61	98.38	98.61	95.84	96.54	96.77	97.23	96.77	97.46	96.77	98.15		85.71	85.18	84.18	84.56
CE1	99.08	99.08	98.85	99.08	96.30	96.77	96.54	97.00	97.23	97.69	97.23	97.00	97.92		87.56	83.56	85.48
CE2	98.85	98.85	98.61	98.85	96.54	97.00	96.30	97.23	97.46	97.92	97.46	97.46	98.15	98.85		84.33	86.48
DAN	98.15	98.15	97.92	98.15	95.38	95.84	95.84	95.61	95.61	97.00	97.00	95.84	97.00	97.92	97.69		84.10
ALAD	98.38	98.38	98.15	98.38	96.30	96.77	96.07	96.54	96.77	97.46	96.77	96.54	97.23	98.38	98.61	97.23	

*Strains were from Lithuania (LT15/164, LT15/174, and LT15/201) and Latvia (LAT). We also used representative strains of Alpe-Adrian (ALAD), Central European (CE), Danish (DAN), Finnish (FIN), North-Scandinavian (N-SCA), Russian (RUS), and South-Scandinavia (S-SCA) lineages. ALAD, FN377821 Hungary; CE1, EU439968 Bavaria; CE2, KT247597 Jura; DAN, AJ238791 Fyn; FIN1, JQ319168 Konnevesi; FIN2, Z30702 Evo; LAT, JN657228 Jelgava1; N-SCA1, AY526219 Umea; N-SCA2, GQ339474 Kiviniemi; S-SCA1, AJ223369 Eidsvoll; S-SCA2, GQ339487 Munga; RUS1, JN657231 Jelgava2; RUS2, JN657232 Madona; RUS3, Z21497 Udmurtia.

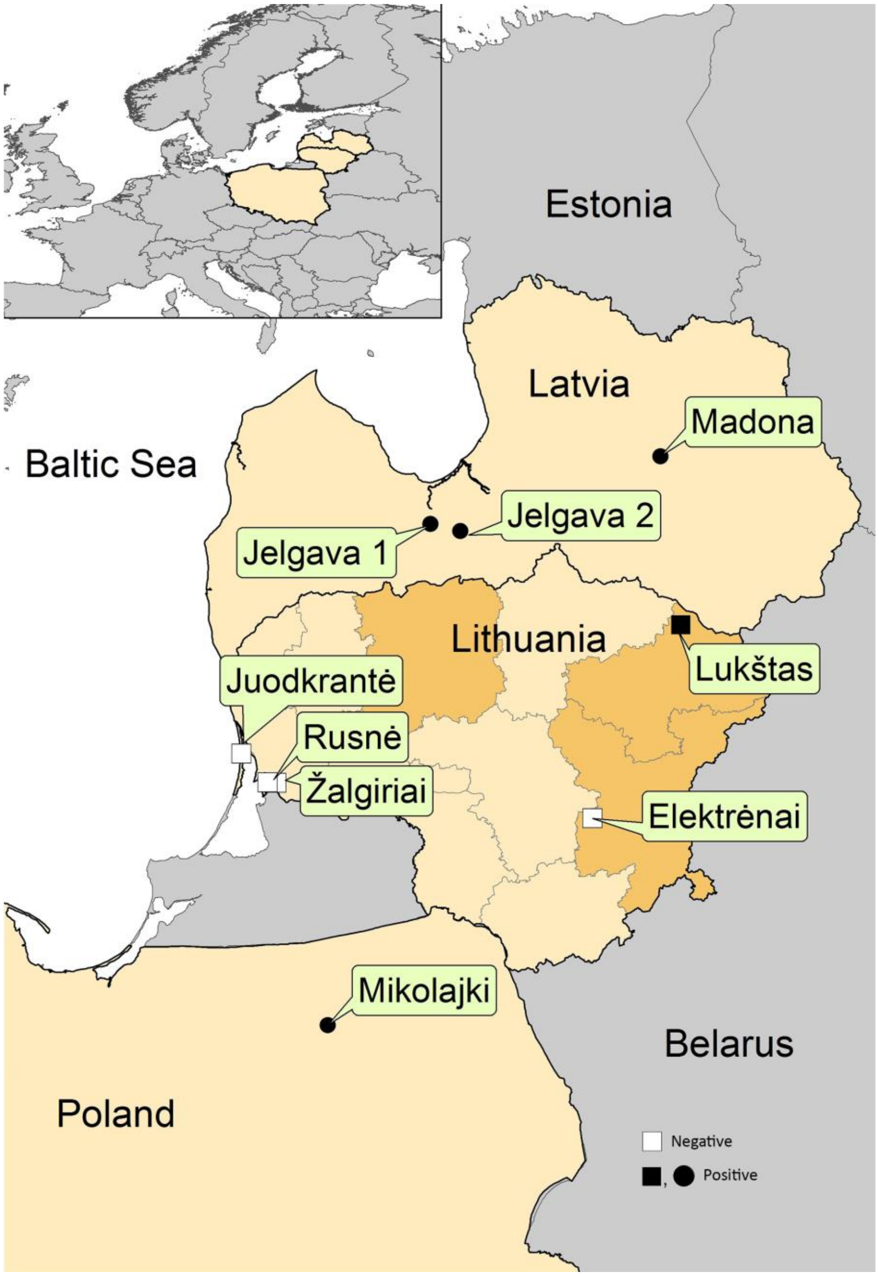

Technical Appendix Figure 1. Lithuania and the surrounding countries (Poland and Latvia) showing 5 trapping sites (squares) for bank voles, which were tested for Puumala virus (PUUV). PUUV-positive localities (Jelgava 1, Jelgava 2, and Madona) in Latvia and (Mikołajki) in from Poland are indicated by circles. For the trapping site in Lukštas, 5 of 45 bank voles were positive for PUUV. At Juodkrantė (n = 28 voles), Elektrėnai (n = 27), Žalgiriai (n = 13), and Rusnė (n = 21), none of the bank voles were positive for PUUV. The 3 counties in Lithuania (Siauliai, Utena, and Vilnius), where previously PUUV-seropositive persons were detected (*1*), are indicated.

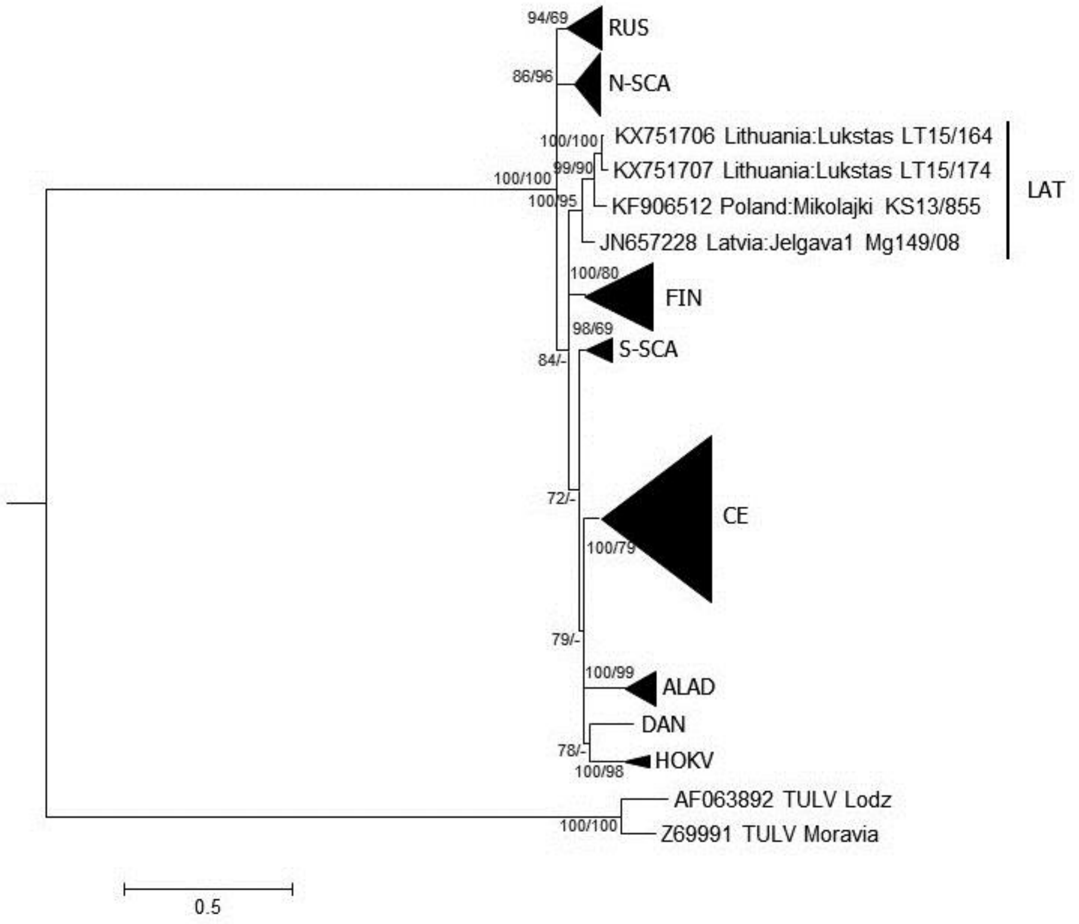

Technical

Appendix Figure 2. Phylogenetic tree based on partial small segment RNA sequences of Puumala virus (PUUV) strains from Lithuania (LT), Latvia (Jelgava1), Poland (Mikołajki), and other PUUV clades. Tula virus (TULV) was used as the outgroup. Phylogenetic calculations were based on Bayesian and maximum-likelihood analyses using MrBayes 3.2.6 (http://mrbayes.sourceforge.net/download.php) and MEGA6 (http://www.megasoftware.net/) with the transition model with invariant sites and gamma distribution and 4,000,000 generations and with the Kimura 2-parameter model and 1,000 bootstrap replicates. A substitution model was determined by using jModelTest 2.1.4 software (https://groups.google.com/forum/#!msg/jmodeltest/qPNGW0K6fdY/Xup7Xy6oAM4J0). Posterior probabilities are indicated before slashes, and bootstrap values are indicated after slashes. Scale bar indicates nucleotide substitutions per site. LT15/165, LT15/166, and LT15/201 were identical to LT15/164 and were therefore excluded from phylogenetic analysis. ALAD, Alpe-Adrian lineage; CE, Central European lineage; DAN, Danish lineage; FIN, Finnish lineage; HOKV, Hokkaido virus; LAT, Latvian lineage; N-SCA, North-Scandinavian lineage; RUS, Russian lineage; S-SCA, South-Scandinavian lineage.

Reference

1. Sandmann S, Meisel H, Razanskiene A, Wolbert A, Pohl B, Krüger DH, et al. Detection of human hantavirus infections in Lithuania. Infection. 2005;33:66–72. PubMed http://dx.doi.org/10.1007/s15010-005-4058-8
